# Child growth monitoring and promotion practice and associated factors among health care workers at public health facilities in south Wollo Zone, Northeast Ethiopia: a facility-based cross-sectional study

**DOI:** 10.1186/s40795-022-00597-6

**Published:** 2022-09-08

**Authors:** Getabalew Getachew Kebede, Yeshimebet Ali Dawed, Kemal Ahmed Seid

**Affiliations:** 1 Dessie Catholic Church School, Dessie, Ethiopia; 2 Department of Nutrition and Dietetics, School of Public Health, College of Medicine and Health Sciences, Wollo University, Dessie, Ethiopia; 3 Department of Public Health, School of Public Health, College of Medicine and Health Sciences, Wollo University, Dessie, Ethiopia

**Keywords:** Child GMP practice, Health care workers, Ethiopia

## Abstract

**Background:**

Growth monitoring and promotion (GMP) is one of the health care priorities to assess and follow the growth pattern of children under 2 years old. Appropriate GMP services enable health care workers to control growth faltering early and child mortality. However, there is limited information showing the practice and associated factors of GMP service among health care workers in Ethiopia. Therefore, this study aimed to assess the practice and identify associated factors of GMP service among health care workers at public health facilities of the South Wollo Zone, northeast Ethiopia.

**Methods:**

A facility-based cross-sectional study was conducted on 397 randomly selected health care workers in the South Wollo Zone, northeast Ethiopia, from May 25 to July 7, 2020. A pretested self-administered questionnaire and in-depth interview were used to collect the quantitative and qualitative data, respectively. Quantitative data were entered using Epi data Version 3.1 and exported to statistical software for social sciences (SPSS) version 20.0 software for further analysis. Binary logistic regression analyses were used to identify factors associated with GMP practice. Statistical tests at a *P* value < 0.05 with a 95% confidence interval were taken as a cutoff point to determine the statistical significance. Qualitative data were analyzed by using thematic analysis.

**Results:**

In this study, the proportion of GMP practice among health care workers was 58.4% (95% CI: 54.0–63.0). Being a holder of first degree (AOR = 2.25; 95% CI: 1.01, 5.05), being a holder of a diploma (AOR = 3.52; 95% CI: 2.04, 6.09), work experience with GMP (AOR = 3.13; 95% CI: 1.58, 6.20), receiving GMP training (AOR = 4.83; 95% CI: 2.89, 8.06), availability of GMP equipment (AOR = 2.75; 95% CI: 1.64, 4.58) and having a positive attitude toward GMP (AOR = 3.70; 95% CI: 2.23, 6.17) were factors significantly associated with GMP practice.

**Conclusions and recommendations:**

The proportion of GMP practice among health care workers was still low. Educational level, work experience with GMP, GMP training, GMP equipment and attitude toward GMP were positively associated with GMP practice. Availability of GMP equipment brings positive attitudes toward GMP, and GMP training for health care workers with less experience should be strengthened to improve GMP practice.

## Introduction

GMP is a preventive and promotive activity comprised of growth monitoring (GM) linked with promotion (P), usually counseling on child feeding and health. It focuses on following up of the growth rate of well-children under 2 years of age in comparison with world health organization (WHO) standards by periodic, frequent anthropometric measurement of weight and plotting it on the weight-for-age growth chart which enables health care workers to see the changes in the children weight and giving counseling to mothers/caregivers about their children growth before children reach the status of malnutrition [1, 2].

Promotional activity relies on the growth pattern of the children. It is used by health care workers to determine appropriate actions to promote children’s growth. These actions include counseling mothers/caregivers about their children’s growth according to their needs and growth pattern [3]. That is why the term promotion was added to growth monitoring to emphasize the action components of GMP activity. GMP, therefore, helps combat child malnutrition through timely and early detection of growth faltering [1]. On the other hand, it helps to reduce children’s mortality, whereby it boosts the achievement of sustainable development goal 3 (SDG3) [4].

To enhance the normal growth and health of children less than 2 years of age, the improvement of GMP practices among health care workers is crucial [5]. Pertaining to GMP coverage and practice, the United Nations International Children’s Emergency Fund (UNICEF) recommends that 100% GMP coverage and practice present a brighter future for children. However, national and international reports have shown that there is a discrepancy between the purpose and the actual practice of GMP. The high prevalence of malnutrition in many developing countries seems to confirm this fact [1, 6, 7]. As several studies indicated since the 1980s, GMP has been promoted as one of the key components of community nutrition programs worldwide for decades [3, 8]. Moreover, a survey on child GMP practices worldwide showed the wide implementation of this routine practice in Asia, Europe, Latin America, and Africa [9]. Moreover, the survey also confirmed that in areas where GMP was implemented as part of a package of nutrition and health programs, positive impacts on child growth outcomes have been reported [9].

Similarly, in Ethiopia, many programmes have been launched to address the issue of persistent malnutrition. Among them, GMP is one of the most effective interventions and forms the basis of comprehensive child care. It has emerged as one of the components of the Ethiopia National Nutrition Strategy (NNS) and National Nutrition Programme (NNP II), and it has been implemented through health facilities at various levels and largely integrated with the health extension package, which aims to stress nutritional counseling, early disease detection and treatment [10, 11]. Health care workers, particularly health extension workers, are the first accountable for GMP service practicality. One of their tasks is to provide GMP service through the “Triple-A” approach” (Assessment, Analysis and Action) [8].

Even though GMP has been practiced for the past 40 years in many countries, including Ethiopia, for millions of children to prevent malnutrition, its practice has been fraught with problems. Its effectiveness has been questioned mostly due to problems in implementation, including low coverage and poor linkage of growth monitoring to promotion activities [1]. Moreover, as reported in a study conducted in Switzerland, problems encountered by health care workers include inadequate interpretation of the growth curve (48%), inaccurate plotting of measurements of weight on the growth chart (40%), and poor understanding of growth reference curves (29%) [9]. Another problem of health care workers in implementing GMP services is inconsistency in performing GMP procedures, which leads them to faulty interpretation of children’s growth patterns, which in turn results in inaccurate information being forwarded to policy makers [6].

Evidence suggests that implementing growth monitoring without linking to the promotion aspect of GMP is a waste of resources and a loss of opportunities [7]. Health care workers mainly emphasized weighing of the children’s weight in the absence of counseling for mothers/caregivers on children’s feeding based on the growth curve [12]. Moreover, studies conducted on GMP practice reported that counseling offered to mothers/caregivers during GMP service is weak [3, 13]. A study conducted in the Tigray Region, Ethiopia, showed that only 16.8% of health care workers offered counseling to mothers/caregivers based on the child growth curve [14].

A study conducted in the Amhara Region, Ethiopia, revealed that the proportion of GMP practice of health care workers was 50.4% [15]. Another study conducted in the Tigray Region, Ethiopia, among health care workers revealed a GMP practice rate of 53.6% [14].

Previous research studies conducted in the country identified factors affecting GMP practice. Attitude, workload, availability of GMP equipment, work experience, and educational level were associated factors that affect GMP practice [15]. Other factors influencing GMP practice were training, supportive supervision and qualification of health care workers [14].

The first 2 years of a child’s life are extremely important. These years have been described as a critical window of opportunity for ensuring appropriate child growth through optimal feeding [16]. GMP, if not practiced appropriately in the first 2 years, can make children more prone to malnutrition [17]. In Ethiopia, although GMP has been implemented since 2008, its practice is not as effective as expected. A study conducted in the Amhara and Tigray regions pointed out that GMP practice is suboptimal. There is still a chance to acquire child malnutrition, and its prevalence is high among children [14, 15].

The 2019 Ethiopia Demography and Health Survey (EDHS) showed that approximately 37, 7 and 21% of children under five were stunted, wasted and underweight, respectively [18].

In a review of the literature, we came across two studies in which one study was conducted in the Amhara Region [15] and the other was conducted in the Tigray Region in the northern part of Ethiopia [14]. The study conducted in the Amhara Region, Ethiopia, focused on growth monitoring practices and their associated factors among health workers but did not focus on the promotion aspect of GMP services [15]. There is also a limited study conducted in Ethiopia in the Amhara region on the practice of GMP services and associated factors among health care workers [6]. Therefore, this study aimed to determine the proportion of GMP practice and identify associated factors among health care workers in the South Wollo zone, Northern Ethiopia. Identifying the determinants of GMP practice helps nutrition program implementers design evidence-based GMP interventions.

## Methods

### Study design, setting and period

A health facility-based cross-sectional study design with both quantitative and qualitative data collection methods was conducted at public health facilities in the south Wollo zone, Amhara Region, Ethiopia, from May 25 to July 7, 2020. The South Wollo Zone has twenty-two districts. According to the Central Statistical Agency (CSA) census of Ethiopia conducted in 2007, the estimated population of the zone is 2,518,862. Of these, 1,248,698 were males, and 1,270,164 were females [19]. The health facilities had trained staff on GMP. GMP service is provided by health care workers for children less than 2 years at the health centers and health post according to the information obtained from the South Wollo Zone Health Department.

### Source population, study population and eligibility criteria

The source populations were all health care workers who have been providing GMP service to children less than 2 years of age in all public health facilities in the South Wollo Zone. The study populations were all health care workers who had been providing GMP service for 1 year and above to children less than 2 years of age in the selected public health facilities in the South Wollo Zone. Those health care workers who had less than 1 year of work experience with child GMP service and those who were on leave were excluded from the study.

### Sample size determination and sampling procedures

The sample size for the quantitative study was calculated by using the single population proportion formula by Epi Info version 7 software by considering the 95% confidence level, 5% margin of error and 50.4% proportion of GMP practice at public health facilities in the North Gondar Zone, Amhara Region, Ethiopia [15]. By adding 10% nonresponse rates, the total number of study participants was 422.

There are 129 governmental health centers and 496 health posts that have GMP services in the South Wollo Zone. Of these, 39 public health facilities (20 health centers and 19 health posts) that provide GMP services were randomly selected and included in the study. The total 422 sample was proportionally allocated for the health centers and health posts based on the number of health care workers in the selected facilities. If there were one or two health care workers in the selected facilities, both were included in the study. However, if more than two HCWs were found, we used the lottery method to select the study participant. To sample respondents from the study population, the list of health care workers in the selected health facilities obtained from the Woreda health office was used as the sampling frame of the study.

After proportional reallocation was employed based on the desired number of health care workers for each selected 39 health facilities, a total sample of 422 health care workers was selected for the quantitative study using a simple random sampling method through random number generation tables. Five interviewees were included in the qualitative study and were purposively selected based on their duration of more service years in providing GMP service for well-children less than 2 years of age.

### Study variables

The dependent variable of the study was child GMP practice. The independent variables of the study were sociodemographic and economic factors (age, sex, educational and marital status, qualification, length of work experience, monthly income, place of work and place of residence), supportive supervision (feedback, report writing, and update the new procedures), training on GMP, workload, logistics and supply (GMP equipment, stationary materials, growth charts, GMPs’ counseling cards.), knowledge and attitude on GMP practice.

### Operational definition

#### Level of knowledge about GMP

To measure the knowledge of the HCW 8 items were used and each correct answer was given a score of 1, and incorrect answers were given a score of 0. Respondents who scored at least 75% of the eleven knowledge questions were labeled “Adequate knowledge” toward GMP practice and those who scored less than 75% were labeled “Inadequate knowledge” toward GMP practice [15].

#### Attitude towards GMP

The attitude questionnaire incorporated 11 items with a five-point Likert scale ranging from 1 (strongly disagree) to 5 (strongly agree). Reverse coding was performed for negatively framed attitude questions. Moreover, the scale was recoded as disagree and agree. Then, each positive response (agree) was given a score of 1 point, and each negative response (disagree) was given a score of 0. Respondents who scored at least 75% of the eleven attitude questions were labeled “Positive attitude” toward GMP practice, and those who scored less than 75% of the eleven attitude questions were labeled “Negative attitude” toward GMP practice [15].

#### Level of GMP practice

To measure the GMP practice 9 questions/items were used and each yes response was given a score of 1 point, and no response was given a score of 0 point.

Finally, the sum was computed for each respondent for practice, attitude and knowledge questions to measure the composite value. Respondents who scored at least 75% of the nine GMP practice questions were labeled “adequate GMP practice”, and those who scored less than 75% of the nine GMP practice questions were labeled “inadequate GMP practice” [15].

#### Level of workload

A health care worker who saw 25 children or more per day was regarded as busy and less than 25 children per day as ideal during GMP sessions [20].

### Data collection procedures

A self-administered pretested Amharic version questionnaire adapted from previous studies was used to collect quantitative data [14, 15]. The data were collected by three data collectors who had experience in collecting data with the support of two supervisors. The questionnaires were distributed by data collectors and self-administered by study participants during the working time. An in-depth interview with a semi-structured interview guide was used to collect qualitative data. The interviewees were asked questions, followed by probes until data saturation was achieved. During the interviews, audio was recorded to prevent data loss and capture the information.

### Data quality assurance

To assure the quality of the quantitative data, the English version of the questionnaire was first translated into Amharic (local language). The questionnaire was pretested using 5% of the total sample size at the Hote Health Center in Dessie City Administration prior to the actual data collection period. Corrections were made for the error, and the problems identified during the pretest survey were solved before the actual data collection activity. One day of intensive training was given for data collectors and supervisors on the overall data collection procedures. Each data collector checked the questionnaires for completeness before distributing them to each study participant. The supervisors conduct on-site supervision during the period of data collection. The completed questionnaires were checked for completeness on a daily basis by supervisors at spot. The principal investigator controlled the overall data collection process on site for the nearby health facilities and with cell phones for long-distance health facilities.

To assure the quality of qualitative data, a semi-structured questionnaire was prepared first in English and then translated into the Amharic version. The audio recorded and notes taken from in-depth interviews were transcribed and translated verbatim. Transcription was performed as soon as possible after the in-depth interviews were conducted to decrease the risk of transcription errors.

### Data processing and analysis

The collected quantitative data were checked for completeness and coded before data entry and entered into Epi data version 3.1 by the principal investigator. For further analysis, the data were exported to SPSS version 20. Descriptive statistics were computed, and the results were reported using frequencies and percentages. Next, bivariable binary logistic regression analysis was performed to determine the association between the outcome variable (GMP practice) and various potential associated factors as independent variables. All independent variables with a *P* value of less than 0.25 in the bivariable binary logistic regression analysis were selected as candidate variables for the multivariable binary logistic analyses. Subsequently, multivariable logistic regression analyses with a backward stepwise (likelihood ratio) method were performed to control the possible effect of confounders and to identify the independent variables that showed significant association with the outcome variable (GMP practice).

Finally, variables with a *p* value less than 0.05 with a 95% confident interval (CI) in the multivariable binary logistic regression model were considered statistically significant determinants of GMP practice. To evaluate the association between GMP practice and each independent variable, both the crude odds ratio (COR) and adjusted odds ratio (AOR) with 95% confidence intervals were reported. The model fitness was checked by using the Hosmer–Lemeshow goodness of the fit test for the model, and the *p* value of the model fitness of the test was 0.28 which is insignificant showing the model is well fitted. Multicollinearity was checked by using variance inflation factors, and all variables had a variance of < 3, showing no multicollinearity. The Cronbach alpha was computed to measure the reliability (internal consistency) between items in the scale for measuring knowledge, attitude and practice towards GMP and was found to be 0.72, 0.76 and 0.81 for knowledge, attitude and practice questions respectively. This result revealed that the items in each measurement scale were reliable and valid to use as a tool to collect the data.

Thematic analysis was used to summarize qualitative data from in-depth interviews. To add the depth of information on GMP practice, the data recorded using audio records and the note taken during the interview were transcribed and translated word by word into English language and then coded and categorized into four different main thematic areas.

## Results

### Sociodemographic and economic characteristics of health care workers

A total of 422 health care workers were targeted, but only 397 participated in the study, giving a response rate of 94.1%. All 397 (100%) of the study participants were females. Of these, 340 (85.6%) were health extension workers. More than one-third 153 (38.5%) of respondents were in the age group of 25–29 years. Of the total study participants, 208 (52.4%) were urban residents, and 220 (55.4%) were diploma holders. Nearly two-thirds 245 (61.7%) of respondents were married, and 358 (90.2%) were from the Amhara ethnic group. Half 197 (49.6%) of the respondents were Orthodox Christians. In terms of workplace, most 342 (86.1%) respondents had been providing child GMP service in the health post. More than three-quarters 317 (79.8%) of respondents had between 1 and 10 years of working experience. More than half 235 (59.2%) of the respondents earned a monthly salary of greater than or equal to 3500 ETB (Table 1).

**Table 1:** Socio demographic and economic characteristics of health care workers in South Wollo Zone, northeast Ethiopia from May 25 to July 7, 2020

Variables	Frequency	Percent
Sex		
Female	397	100%
Age groups		
20–24	79	19.9
25–29	153	38.5
30–34	88	22.2
≥ 35	77	19.4
Place of residence		
Urban	208	52.4
Rural	189	47.6
Marital status		
Married	245	61.7
Currently unmarried	152	38.3^a^
Qualification		
Nurse	57	14.4
Health extension worker	340	85.6
Educational level		
Certificate	128	32.2
Diploma	220	55.4
First degree	49	12.3
Religion		
Muslim	184	46.3
Orthodox	197	49.6
Catholic	6	1.5
Protestant	10	2.5
Ethnicity		
Amhara	358	90.2
Oromo	36	9.1
Tigre	3	0.8
Work place		
Health center	55	13.9
Health post	342	86.1
Work experience		
1–10	317	79.8
≥ 11	80	20.2
Income per month (ETB)		
1500–2499	13	3.3
2500–3499	149	37.5
≥ 3500	235	59.2

^a^ Currently unmarried includes single, divorced, separated and widowed, *ETB* Ethiopian Birr

### Availability of GMP equipment, training and supportive supervision

Nearly two-thirds (255, 64.2%) of respondents reported that they had GMP equipment in their health facilities. Less than two-thirds of the respondents (235, 59.2%) replayed that they had taken GMP training. The most common training types received by respondents were weight skill (224, 40.1%), the plotting technique of growth chart (219, 39.2%) and child feeding counseling (116, 20.8%). More than three-quarters 231 (78.0%) of the training was provided by district health office, and less than half 185 (46.6%) of them took the training from 1 to 10 times. Nearly half (52.6%) of the respondents had received supportive supervision, and nearly three quarters 198 (77.3%) of the supportive supervision was provided by district supervisors (Table 2).

**Table 2:** Availability of GMP equipment, training and supportive supervision for health care workers in South Wollo Zone, northeast Ethiopia from May 25 to July 7, 2020

Variables		Frequency	Percent
Availability of equipment to do GMP			
Yes		255	64.2
No		142	35.8
Training attended by HCW on child GMP			
Yes		235	59.2
No		162	40.8
Training attended			
on weigh skill^a^	Yes	224	40.1%
on plotting technique^a^	Yes	219	39.2%
on child feeding counseling^a^	Yes	116	20.8%
Training provided			
by District health office^a^	Yes	231	78.0%
by NGO^a^	Yes	65	22.0%
Number of training taken by HCWs			
1–10		185	46.6
≥ 11		50	12.6
Supportive supervision on child GMP			
Yes		209	52.6
No		188	47.4
Supportive supervision			
by District supervisory ^a^	Yes	198	77.3%
by NGO^a^	Yes	58	22.7%

^a^Multiple responses, *GMP* Growth Monitoring and Promotion, *HCW* Health Care Worker, *NGO* Non-Governmental Organization

### Workload

Half 201 (50.6%) of the respondents reported that they had seen fewer than 25 children to follow during GMP sessions per day. More than half 222 (55.9%) of the participants took an average of one to 10 min for one child to assess GMP. Nearly three quarters 309 (77.8%) of the respondents had another work in addition to GMP (Table 3).

**Table 3:** Workload for health care workers in South Wollo Zone, northeast Ethiopia from May 25 to July 7, 2020

Variables	Frequency	Percent
Number of children seen during GMP session per day		
< 25	201	50.6
≥ 25	196	49.4
Average time in minute to assess GMP for one child		
1–10	222	55.9
≥ 11	175	44.1
Another work in addition to GMP		
Yes	309	77.8
No	88	22.2

*GMP* Growth Monitoring and promotion

### Knowledge of health care workers on GMP practice

A large percentage 322 (81.1%) and 365 (91.9%) of the respondents knew the purpose GMP and the benefit of the growth chart, respectively. Three hundred thirty-one (83.4%) respondents knew that children between 0 and 2 years should be weighed every month. Most 361 (90.9%) respondents understood the meaning of the rising plotted line on the growth chart. Regarding the meaning of a falling plotted line on the growth chart, 354 (89.2%) respondents knew the meaning. More than half 229 (57.7%) of respondents explained a plotted line that deviates upward above the upper limit of the normal as excess gain of weight. Almost three quarters 295 (74.3%) of respondents knew that downward deviation of the plotted line below the lower limit of normal indicates inadequate weight gain. Seventy-five percent of the participants knew the importance of starting complimentary foods at the age of 6 months in addition to breast milk. Of the total study participants, 265 (66.8%) and 233 (58.7%) of the respondents knew the recommended daily feeding frequency for 6–8-month-old breastfeeding children and for 9–23-month-old breastfeeding children, respectively. However, for the nonbreastfeeding 6–23-month-old children, only 160 (40.3%) of the respondents knew the recommended daily feeding frequency. Concerning the overall knowledge score of the participants, 328 (82.6%) had adequate knowledge about GMP services (Table 4).

**Table 4:** Knowledge of health care workers towards GMP practice in South Wollo Zone, northeast Ethiopia from May 25 to July 7, 2020

Variables	Frequency	Percent
Purpose of GMP		
Know	322	81.1
Don’t know	75	18.9
Benefit of Growth chart		
Know	365	91.9
Don’t know	32	8.1
Frequency of weighing schedule for children aged group 0–2 years		
Know	331	83.4
Don’t know	66	16.6
Meaning of a plotted line rising on the growth chart		
Know	361	90.9
Don’t know	36	9.1
Meaning of a plotted line falling on the growth chart		
Know	354	89.2
Don’t know	43	10.8
Indication of deviations of plotted line above the upper reference curve		
Know	229	57.7
Don’t know	168	42.3
Indication of deviations of plotted line below the lower reference curve		
Know	295	74.3
Don’t know	102	25.7
Reason for giving complementary foods at six months of age		
Know	298	75.1
Don’t know	99	24.9
Recommended daily feeding frequency for 6–8 month old breastfeeding child		
Know	265	66.8
Don’t know	132	33.2
Recommended minimum daily feeding frequency for 9–23 month old breastfeeding child		
Know	233	58.7
Don’t know	164	41.3
Recommended daily feeding frequency for 6–23 month old non breastfeeding child		
Know	160	40.3
Don’t know	237	59.7
Knowledge Status		
Adequate knowledge	328	82.6
Inadequate knowledge	69	17.4

*GMP* Growth Monitoring and promotion

### Attitudes of health care workers toward GMP practice

More than two-thirds (283, 71.3%) of respondents believed that GMP is important for the well-being of every child, and nearly three-quarters (294, 74.1%) of the respondents believed that GMP is effective in preventing malnutrition. Of the 397 respondents, 292 (73.6%) believed that regular monthly weighing of weight is important. However, 346 (87.2%) of the respondents considered the process of child GMP to be time consuming, and similarly, more than three-quarters 322 (81.1%) of the respondents considered the process of GMP to be burdensome. Nearly three quarters, 313 (78.8%) and 338 (85.1%) of the respondents disagreed that weighing the weight did not make them happy and that the process of GMP was inconvenient, respectively. Three hundred eight (77.6%) of the respondents believed that mothers/caregivers should be involved in child GMP sessions, and three quarter 263 (66.2%) of the respondents agreed that counseling of mothers/caregivers makes GMP complete. Approximately 284 (71.5%) of the respondents believed that the trainings had been enhancing health care workers to conduct GMP effectively, and 276 (69.5%) of the respondents agreed that supportive supervision for health care workers is important for GMP practice. In general, when an overall attitude score was computed, 249 (62.7%) of the respondents had a positive attitude toward GMP service (Table 5).

**Table 5:** Health care workers attitude towards GMP practice in South Wollo Zone, northeast Ethiopia from May 25 to July 7, 2020

Variables	Frequency	Percent
GMP is necessary for the well-being of every children		
Agree	283	71.3
Disagree	114	28.5
Regular monthly weighing of child weight is important		
Agree	292	73.6
Disagree	105	26.4
Weighing of the child weight make you happy		
Agree	84	21.2
Disagree	313	78.8
The process of child GMP is time consuming		
Agree	346	87.2
Disagree	51	12.8
The process of child GMP is convenient		
Agree	59	14.9
Disagree	338	85.1
Child GMP is effective in preventing childhood malnutrition		
Agree	294	74.1
Disagree	103	25.9
Mothers/caregivers should be involved in child GMP sessions		
Agree	308	77.6
Disagree	89	22.4
Process of child GMP is burdensome.		
Agree	322	81.1
Disagree	75	18.9
Counseling of mothers/caregivers makes child GMP complete		
Agree	263	66.2
Disagree	134	33.8
Training enhances HCW ability to do child GMP effectively		
Agree	284	71.5
Disagree	113	28.5
Supportive supervision important for child GMP practice		
Agree	276	69.5
Disagree	121	30.5
Attitude Status		
positive attitude	249	62.7
Negative attitude	148	37.3

*GMP* Growth Monitoring and Promotion, *HCW* Health Care Worker

### Health care workers’ GMP practice

Almost all 396 (99.7%) of the respondents took the child weight monthly while doing GMP in their health facilities. Most 350 (88.2%) of the respondents would undress/light dress the child before weighing the child weight. Almost half 196 (49.4%) of the respondents checked the accuracy of the weight scale, and more than half 248 (62.5%) of the respondents adjusted the weight scale to zero with an empty plastic washing bowl before weighing the child weight. Nearly two-thirds 268 (67.5) of the respondents plotted the growth curve on the growth chart while doing GMP, and more than half 225 (56.7) and almost half 202 (50.9%) of the respondents were also able to interpret the growth curve and explain it to mothers/caregivers while doing GMP, respectively. Less than half 167 (42.1%) of the respondents counseled mothers/caregivers based on the growth curve when carrying out GMP. Almost all 382 (96.2%) of the respondents advised mothers/caregivers to introduce only breast milk for 6 months and solid, semisolid or soft foods at 6 months for their child in addition to breast milk. Although 328 (82.6%) respondents had adequate knowledge and 249 (62.7%) of the respondents had a positive attitude toward GMP, the overall proportion of GMP practice among health care workers was 58.4% (95% CI: 54.0–63.0) (Table 6).

**Table 6:** Health care workers GMP Practice in South Wollo Zone, northeast Ethiopia from May 25 to July 7, 2020

Variables	Frequency	Percent
Weigh the child weight while doing growth monitoring and promotion		
Yes	396	99.7%
No	1	0.3
Check the accuracy of the weight scale while doing GMP		
Yes	196	49.4
No	201	50.6
Undress/ light dress the child while weighing the child weight		
Yes	350	88.2
No	47	11.8
Adjust the weight scale to zero with plastic washing bowl before weighing the child weight		
Yes	248	62.5
No	149	37.5
Explain growth curve to mothers/caregiver while doing GMP		
Yes	202	50.9
No	195	49.1
Plot the growth curve on growth chart while doing GMP		
Yes	268	67.5
No	129	32.5
Interpret the growth curve while doing GMP practice		
Yes	225	56.7
No	172	43.3
Counsel mothers/caregivers based on the growth curve when carrying out GMP		
Yes	167	42.1
No	230	57.9
Counsel mothers/caregivers when to introduce breast milk and solid, semi-solid for their child		
Yes	382	96.2
No	15	3.8
Practice status		
Good GMP practice	232	58.4
Poor GMP practice	165	41.6

*GMP* Growth Monitoring and promotion

### Factors associated with the GMP practice of health care workers

In this study, the results of bivariable binary logistic regression analysis found that the educational level of health care workers (being a diploma and first degree holder), length of work experience on GMP, GMP training, Supportive Supervision on GMP, availability of GMP equipment and attitude of health care workers toward GMP had significant associations with the outcome variable with a *P* value of < 0.2.

In the multivariable binary logistic regression, being a first-degree holder health care worker (AOR = 2.25; 95% CI: 1.01, 5.05), being a diploma holder (AOR = 3.52; 95% CI: 2.04, 6.09), having work experience ≥11 years (AOR = 3.13; 95% CI: 1.58,6.20), taking training on GMP (AOR = 4.83; 95% CI: 2.89, 8.06), having GMP equipment (AOR = 2.75; 95% CI: 1.64, 4.58), and having a positive attitude toward GMP (AOR = 3.70; 95% CI: 2.23, 6.17) had a statistically significant association with GMP practice with *p* value< 0.05 (Table 7).

**Table 7:** Factors associated with GMP practice among health care workers in South Wollo Zone, northeast Ethiopia from May 25 to July 7, 2020

Variables	GMP Practice		COR (95% CI)	AOR (95% CI)	*P*-value
	Good	Poor			
Education level					
First degree	31	18	2.777(1.405–5.489)	2.255(1.005–5.059)	.049*
Diploma	152	68	3.604(2.282–5.691)	3.529(2.044–6.093)	.0001*
Certificate	49	79	1	1	
Work experience on GMP					
≥ 11	62	18	2.978(1.686–5.263)	3.134(1.584–6.202)	.001*
1–10	170	147	1	1	
Training attended on GMP					
Yes	177	58	5.937(3.823–9.219)	4.831(2.894–8.065)	.0001*
No	55	107	1	1	
Supportive Supervision on GMP					
Yes	155	54	4.138(2.706–6.328)	1.428(.813–2.510)	.215
No	77	111	1	1	
Availability of GMP equipment					
Yes	154	101	3.280(2.163–4.974)	2.751(1.648–4.589)	.0001*
No	78	64	1	1	
Attitude on GMP					
Positive	175	74	3.775(2.460–5.794)	3.709(2.228–6.174)	.0001*
Negative	57	91	1	1	

*Significant at *p*-value < 0.05, *GMP* Growth Monitoring and Promotion, *COR* Crude odds ratio, *AOR* Adjusted odds ratio

### Interview

An in-depth interview was carried out with a total of five interviewees who provided GMP service in their health facility for children under 2 years of age. They have eight and above years of work experience in providing child GMP service. The main themes that emerged from interviews with health care workers were child feeding counseling, attitude toward GMP, training on GMP and resources for GMP. The key findings are organized under different thematic areas and presented below.

### Child feeding counseling

The majority of the interviewed participants said that they offered counseling to mothers/caregivers. During the GMP session, they said that they gave more emphasis to infant breastfeeding and complementary feeding. Few said that they were so busy and the counseling time was too short to discuss more with mothers/caregivers as a result of which they believed that they were not performing the counseling appropriately.

A 29-year-old rural health extension worker with 9 years of experience in GMP practice said, “*… As we do the growth monitoring, there is also child feeding counseling that goes out to the mothers/caregivers. We gave advice to the mothers/caregivers about child breastfeeding and complementary feeding. Regarding breast feeding, we told them to provide only breast milk for their child until their children reached six months. We also provided advice on what foods they should give (solid, semisolids, liquid) when the child is above six months in addition to breast milk.”*

### Attitudes toward GMP practice

Most of the interviewed participants believed that GMP is one of the different important tools used to advance the normal growth of children. They also looked upon it as effective as to reduce malnutrition. Some of the participants pointed out that it is very helpful in providing information on whether a child grows well or not.

A 32-year-old urban health extension worker with 10 years of experience in GMP practice said, *“… GMP is a good and important tool, easy to use and effective. It is one of the services that we have to do to reduce child malnutrition.”*

### Training on GMP practice

One of the interviewed participants reported that training on GMP service is important. However, counseling of mothers/caregivers on child feeding and health is given partially due to lack training on child feeding counseling.

A 29-year-old rural health extension worker with 9 years of experience in GMP practice said *“…*. *Even if we have been trained, the training is not adequate. In-service training in GMP service is important, if there are changes, every time there is a different way of doing things, to keep up the standard and to update the procedures of GMP training is important.”*

### Resource for GMP practice

More than half of the interviewed participants reported that they were often understaffed. In addition, there are activities that they do in line with GMP, such as immunizations and family planning, which altogether result in a heavy workload. Regarding equipment/supply, almost more than half of the participants reported that there was a lack of equipment and supply, such as growth charts, registration books, visual aids and referral formats.

A 35-year-old urban nurse with 11 years of experience in GMP practice said, “… *sometime a large number of children that we are seeing during GMP sessions result in a heavy workload. Because we are understaff. We are in need of more health care workers. If we had more staff, I think it would be better to do GMP.”*

A 30-year-old rural health extension worker with 8 years of experience in GMP practice said, *“… There is a lack of GMP equipment and shelter to perform GMP. When we have everything at hand, then everything works on GMP well.”*

## Discussion

This health facility-based cross-sectional study was performed to assess child GMP practices and associated factors among health care workers who provided GMP services at public health facilities in the South Wollo Zone, Amhara Region, Ethiopia.

The findings of this study showed that the overall proportion of good child GMP practice was 58.4% (95% CI: 54.0–63.0). This indicates that the proportion of good child GMP practice was slightly higher than reports from the North Gonder Zone, Amhara Region (50.4%) [15] and the Tigray region, Ethiopia (53.6%) [14]. The possible reason for this variation might be the difference in the study setting. This may also be attributable to the time difference, as there could be current improvement in accessing and practicing GMP services over time.

In multivariable binary logistic regression, first-degree holder health care workers had a positive association with GMP practice. The odds of GMP practice among first-degree holder health care workers were two times higher compared to the odds of GMP practice among certificate holder health care workers, which is in line with a study conducted in Bahir Dar Health centers [21]. The possible reason might be that those degree holders have longer exposure to GMP practice in their under graduate clinical attachment periods. However, a study from the North Gondar Zone, Amhara Region, Ethiopia revealed no significant association between having a first degree and GMP practice [15]. The possible reason for this difference could be related to the participants in the previous study being direct university graduates who might not have taken in-service training on GMP practice before the time of data collection. Similarly, diploma holder health care workers had a positive association with GMP practice. This study revealed that the odds of GMP practice among diploma health care workers were almost four times higher than the odds of GMP practice among certificate health care workers. A study from North Gondar Zone, Amhara Region, Ethiopia reported that diploma health care workers have a positive association with GMP practice [15]. The possible explanation for the difference between first-degree and diploma health care workers with certificate health care workers in terms of GMP practice might be their educational background and experience. This means that first-degree and diploma holder health care workers might have a first-hand chance to be exposed to any training, including GMP practices and experience sharing conferences, which made them more aware of GMP services than those who had certificates to utilize available GMP equipment and provide GMP services. Therefore, health extension workers (certificate holders) need to be empowered (obtain learning opportunities) to improve the quality of GMP service delivery.

The other factor that was positively associated with GMP practice of health care workers in this study was length of work experience. The odds of GMP practice among health care workers who had work experience ≥11 years were three times higher than the odds of GMP practice among health care workers who had work experience 1–10 years. This result is in line with the study conducted in the Tigray region, North Gonder Zone and Bahir Dar, Amhara region, Ethiopia [14, 15, 21]. A possible explanation might be that increased work experience enables health professionals to be exposed to GMP practice and perform the procedures well.

In the present study, the availability of GMP equipment in health facilities was positively associated with GMP practice. The odds of GMP practice of the health care workers who had access to GMP equipment were almost three times higher compared to the odds of GMP practice of health care workers who did not have GMP equipment. This is supported by a study performed in the North Gondar Zone and Bahir Dar, Amhara Region, Ethiopia [15, 21]. This result was also supported by the results obtained from the interview part of this study.

*“… There is lack of GMP equipment and shelter to perform GMP. When we have everything at hand then everyone works on GMP well.”* (A 30-year-old rural health extension worker).

A possible explanation might be that the existence of adequate equipment enables them to practice GMP and provides job satisfaction compared to those who had no adequate equipment in their health facilities.

A positive association between health care workers with positive attitudes and their GMP practices was also observed in this study. The odds of GMP practice among health care workers who had a positive attitude toward GMP were four times higher than the odds of GMP practice among health care workers who had a negative attitude toward GMP, in line with a study conducted in South Africa.

and Bahirdar [20, 21] This is also supported by the findings from the qualitative (interview) part of the study.

*“… GMP is a good and important tool, easy to use and effective. It is one of the services that we have to do to reduce the number of children under nutrition*. (A 32-year-old urban health extension worker).

The possible reason for this might be that having a positive attitude leads to better utilization of a growth chart there by increasing the chance of practicing the GMP activity. This implies that the attitude of health care providers toward the importance of GMP practice is one of the critical issues to be considered by the concerned bodies.

In this study, a positive association was also found between attending training on GMP and GMP practice. The odds of GMP practice of the health care workers who took training on GMP were five times higher compared to the odds of GMP practice of the health care workers who did not take training on GMP. The results of studies conducted in Nepal and Ethiopia showed a positive association, supporting the results of this study [21, 22].

This result was also supplemented by the result obtained from the interview.

*“…. Even if we have been trained, the training is not adequate. In-service training on GMP service is important, if there are changes every time there is a different way of doing things, to keep up the standard and to update the procedures of GMP, training is important”* (A 29-year-old rural health extension worker)*.* A possible explanation might be that health professionals who received training usually acquired technical skills, interpersonal communication skills, and solid knowledge to perform their jobs efficiently in the workplace.

The practical implication of this finding is that GMP practice is clearly related to health care workers’ educational level, work experience, training, positive attitude toward GMP and availability of GMP equipment. Health care workers who have a first degree and diploma, higher work experience on GMP, training on GMP, and a positive attitude toward GMP are more likely to practice GMP. It will be used as an input for the national nutritional strategy, the national nutritional program of Ethiopia and the Thin on the Ground (Save the Children UK in Ethiopia) to reduce malnutrition. In addition, it is an input for declarations that were planned by the government of Ethiopia, such as Seqota declarations, to end up malnutrition problems.

### Strength and limitations of the study

As this study used a cross-sectional study design, it is limited to showing cause-and-effect relationships between GMP practice and potential independent factors. Unfortunately, in this study, all the participants were females, which could also affect the findings and was taken as another limitation. Despite these limitations, a mixed approach (qualitative and quantitative) is used to supplement the results and to explore factors that are not addressed by quantitative methods.

## Conclusions and recommendations

In conclusion, the overall proportion of good child GMP practice among health care workers in the South Wollo Zone was still low compared to the UNICEF recommendation that 100% GMP coverage and practice is essential. Level of education, work experience on GMP, training on GMP, availability of GMP equipment and positive attitude toward GMP were positively associated with GMP practice among health care workers. Therefore, the Ministry of Health should strengthen the availability of GMP equipment, training on GMP, and bring positive attitudes toward GMP using motivational strategies to improve the GMP practice of health care workers, with a special focus on those with less experience in GMP service. Growth monitoring and promotion should be emphasized by implementing a good training package, availing adequate growth monitoring equipment, and providing educational opportunities for certificate holders.

## Data Availability

All the required data have been included in the manuscript and its supplementary information files. The datasets used and/or analyzed during the current study are available from the corresponding author on reasonable request.
